# Primary health care response to tuberculosis treatment in Brazilian cities during the COVID-19 pandemic: a mixed-method study

**DOI:** 10.1080/16549716.2025.2556529

**Published:** 2025-09-18

**Authors:** Roxana Isabel Cardozo Gonzales, Daiane Cardoso da Silva, Johannes Abreu de Oliveira, Hellen Cristina Sthal, Paula Hino, Sabrina da Silva de Souza, Maria Rita Bertolozzi, Roberta Ramos Ribeiro, José Luís Guedes dos Santos, Stephanie Ribeiro, Claudia Susana Pérez Guerrero, Karlla Antonieta Caetano, Sheila Araújo Teles, Tânia Maria Ribeiro Monteiro de Figueiredo, Juliana Soares Tenorio de Araújo

**Affiliations:** a Faculdade de Enfermagem, Universidade Federal de Goiás, Goiânia, Brazil; b Escola Paulista de Enfermagem, Universidade Federal de São Paulo, São Paulo, Brazil; c Departamento de Enfermagem, Universidade Federal de Santa Catarina, Florianópolis, Brazil; d Escola de Enfermagem, Universidade de São Paulo, São Paulo, Brazil; e Faculdade de Enfermagem, Universidade Estadual da Paraíba, João Pessoa, Brazil; f Faculdade de Enfermagem, Universidade Estadual de Ciências da Saúde de Alagoas, Maceió, Brazil

**Keywords:** tuberculosis, therapeutics, Covid-19, primary health care, public health

## Abstract

**Background:**

The interruption of tuberculosis care and monitoring activities during the Covid-19 pandemic resulted in delays in diagnosis and treatment of this disease, which compromised progress towards the goal of elimination.

**Objectives:**

Analyze tuberculosis-related activities offered in primary health care settings in Brazil during 2020–2022

**Methods:**

This mixed-method convergent parallel study was conducted in four state capitals, with the number of health units defined by sample calculation. Professionals in various areas were interviewed as key informants in primary care services to investigate tuberculosis-related activities provided during the pandemic. Using these findings, we identified common themes in both the quantitative and qualitative data

**Results:**

Four major themes were identified: ‘Consultations for people undergoing tuberculosis treatment within the context of health reorganization;’ ‘Compromised testing and surveillance;’ ‘Drug dispensing in collaborative activities to reduce the exposure of people undergoing treatment;’ and ‘Changes in directly observed therapy to reduce infection risk.’ Changes in the tuberculosis-related activities were identified in all four cities during the study period; the mean rates of change were lowest in São Paulo and higher in Goiânia and João Pessoa for nearly all the activities offered.

**Conclusions:**

Structural barriers must be identified in each city (such as laboratory network function, reorganization strategies, and local and national directives) to address specific needs related to tuberculosis care during emergency situations and continue progress toward eliminating this disease.

## PAPER CONTEXT

- **Main findings**: The Covid-19 pandemic interrupted tuberculosis-related activities in the four Brazilian cities we analyzed (particularly Goiânia and João Pessoa), highlighting structural inequalities in the primary health care system.
- **Added knowledge**: This study integrated quantitative and qualitative analyses to reveal how the Covid-19 pandemic affected tuberculosis care and monitoring, offering insights into systemic barriers faced in primary care services in Brazil.
- **Global health impact for policy and action**: Stronger laboratory networks and continuity of care policies are critical to prevent interruptions in essential tuberculosis treatment services during global health emergencies.

## Background

Tuberculosis (TB) remains a major public health problem globally, especially in countries with limited resources [1]. Due to its epidemiological magnitude as well as its economic and social repercussions, elimination of this disease is included in the United Nations Sustainable Development Goals and the Global End TB Strategy, which sets a target of eradicating TB as a public health problem by 2035 [2]. In line with global policy, Brazil established its National Plan to End TB in 2017. But since 2020, both the epidemiological scenario of TB and operating conditions of health services in Brazil and worldwide have deteriorated as a result of the Covid-19 pandemic [3,4]. Between 2018 and 2022, a total of 34 million people around the world were treated for TB, below the target of 40 million set by the Global TB Elimination Plan [5]. Although TB treatment is offered free of charge in Brazil by the Unified Health System (the *Sistema único de Saúde*, known as the SUS), in 2022 the cure rate was 59.6% [4]; this result was impacted by the Covid-19 pandemic [6], which has had negative repercussions on adherence to treatment, case monitoring, and continuity of health services, especially in primary health care (PHC).

Studies investigating the pandemic period have found reduced numbers of notified cases and people initiating TB treatment [7], as well as losses to follow-up among people undergoing treatment [8]. They also observed fewer visits to health services by users undergoing treatment [9] and decreases in home visits to health service users with TB [10], as well as in supply and patient receipt of TB drugs [9,11]. On the other hand, medication dispensing for longer periods became more frequent [10], as users faced difficulties in both accessing medicines [12] and treatment for this disease [11,13]. Increases were also seen in retreatment [14] and treatment discontinuation in cases of resistant TB [11]. Around the world, PHC services had to change their care routines [10,11,15–17], which compromised their ability to identify cases early and start treatment in a timely manner and also address health problems and needs [18]. Health services had to restructure to meet new care requirements in response to the Covid-19 pandemic. This led to significant changes in everyday PHC routines, especially as health professionals were reallocated, which had a direct impact on the treatment dynamics for people with TB [19,20]. In many services, TB care (especially within the context of PHC) was even more neglected [21–23].

The Pan American Health Organization emphasizes the central role of PHC, especially in terms of direct community access to health services. The role of PHC in reducing the global burden of disease [24] and attaining goals in the elimination of TB as a public health problem is widely recognized [25]. TB management activities are extensively carried out in local healthcare facilities, known as PHC units, which serve as the main non-emergency entry points to care within Brazil’s Unified Health System (SUS). These units play a critical role in the prevention, diagnosis, and treatment of the disease, in alignment with the organization of the national healthcare network [26].

Within this context, this study investigated the repercussions of the Covid-19 pandemic on tuberculosis treatment and monitoring activities in PHC in four Brazilian capital cities during the pandemic period (2020–2022), analyzing changes in availability.

## Methods

### Study design

This is a multi-center study with a convergent parallel mixed-method design (CRESWELL; CRESWELL, 2022). The quantitative approach is a cross-sectional study based on primary data that made it possible to estimate the magnitude and pattern of repercussions of Covid-19 on TB care and monitoring activities. The qualitative, descriptive approach allowed us to deepen our understanding of these repercussions from the perspective of healthcare professionals who were considered key informants in the PHC services. To ensure methodological rigor, we followed the recommendations of the Mixed Methods Appraisal Tool (MMAT) [27].

### General setting

**Figure 1.**
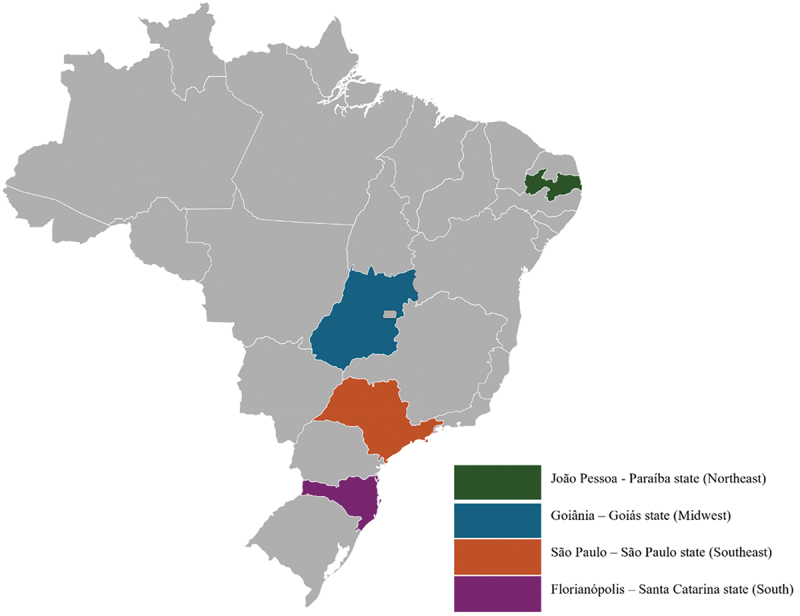
Brazilian cities studied [28].

This study examined PHC services in urban areas of four Brazilian state capitals: João Pessoa, Goiânia, São Paulo and Florianópolis. These four cities are located in different regions of the country (Figure 1), and were selected by convenience, based on existing institutional partnerships.

The cities differ in terms of their socioeconomic aspects, PHC structure and TB epidemiological indicators, as shown in Table 1. They range in population from approximately 500,000 in Florianópolis to over 11 million in São Paulo. Their Human Development Index (HDI) rankings are very high to high, with per capita gross domestic product (GDP) ranging from USD 11,763.86 in São Paulo to USD 4,738.55 in João Pessoa [30]. In 2024, São Paulo and João Pessoa had the highest incidences of TB per 100,000 inhabitants (61 and 55.1 cases, respectively); while Florianópolis (38.2) was closest to the national indicator (39.7); Goiânia had the lowest rate of the four cities analyzed (13.4 cases), one of the lowest in the country [4].

**Table 1.** Characteristics of analyzed cities: demographic, socioeconomic, primary health care and tuberculosis-related data.

Location	Population	HDI	GDP	Percentage of PHC coverage	Number of PHC units	Number of new TB cases	TB incidence rate (per 100,000 inhabitants)	TB mortality rate (per 100,000 inhabitants)
São Paulo	11,451,999	0.806	USD 11,763.86	47.09%	468	7252	61	1.1
Goiânia	1,437,366	0.799	USD 6,769.78	50.63%	82	200	13.4	0.7
João Pessoa	833,932	0.763	USD 8,022.19	79.95%	93	490	55.1	8.8
Florianópolis	537,211	0.847	USD 4738.55	94.28%	51	220	38.2	6

Source: Adapted from [4,29,30].

HDI: Human Development Index; GDP: gross domestic product; PHC: primary health care; TB: Tuberculosis.

### Tuberculosis care in the Brazilian health system

The National Tuberculosis Control Policy (PNCT) establishes guidelines for tackling TB as a public health problem in Brazil, and incorporates epidemiological surveillance, early diagnosis, free treatment, medication dispensing and case monitoring through the national health care system [29].

Treatment is offered universally and free of charge, with an emphasis on directly observed therapy (DOT) and standardized protocols according to the guidelines of the Manual of Recommendations for TB Control in Brazil [30] (based on the World Health Organization directives) [31,32] to ensure patient adherence and minimize treatment interruptions [29].

The Brazilian SUS public health system was established by the 1988 Federal Constitution to guarantee the entire population universal, comprehensive and free access to health care. Based on the principles of universality, equity and comprehensiveness, the system ensures that everyone has the right to care without discrimination and that complete services are provided according to each individual’s needs. It ranges from primary services with health promotion and disease prevention activities to specialized services such as rehabilitation and palliative care, urgent and emergency care, surgeries, transplants and free distribution of medications [33].

The system is funded jointly by federal, state and municipal governments; the federal government transfers most of the funding to the states and municipalities, which are required to invest a minimum percentage of their budgets in health care (15% for municipalities and 12% for states). These resources are organized into funding blocks and distributed according to criteria such as population served, specific local needs, and targets established with federal agencies [34].

Primary care services in Brazil are predominantly organized around the family health strategy [35]. Unlike the traditional model, its focus extends beyond the individual and considers the family as the unit for providing care. Family health teams are comprised of professionals from different disciplines and include at least one doctor, one nurse, one nursing technician and community health workers. Their work is based on integrating different types of knowledge in order to understand and intervene in the multiple determinants that influence the health-disease process [34]. All PHC teams offer TB diagnosis and treatment services.

Note that this study did not include patients linked to private health insurance plans, since TB treatment in Brazil is offered free of charge via the SUS.

### Sample size and study participants

A probabilistic sample was calculated to define the number of PHC units participating in the study, based on the estimated prevalence for each outcome. The following formula was used, where *N* represents the total number of health units in the municipality and the estimated proportion of the outcome, and *d* represents the absolute estimation error (or precision). The proportion value (p) was set at 50%, since this value maximizes the sample size.

$$n=\frac{{z^{2}}_{\alpha /2}\mathrm{Np}\left(1-p\right)}{d^{2}\left(N-1\right)+{z^{2}}_{\alpha /2}p\left(1-p\right)}$$

The PHC services were selected by stratified random sampling according to the organizational structure of each location (district, coordination or zone), with proportional allocation so the number of health units selected in each region was proportional to the total number of units and the population of each stratum. A total of 81 health units were selected in São Paulo, 48 in Goiânia, 48 in João Pessoa and 32 in Florianópolis. Although this sampling method is intended to ensure the representativeness of different regions, it is important to note that proportional allocation may result in the selection of more PHC services in areas with a higher population density; while this ensures more complete coverage of these locations, it may also disregard the vulnerability of less populous regions with greater difficulties accessing health services. Furthermore, the selection of PHC services using this approach can also be influenced by the administrative and logistical organization of health services, which in some cases can result in a bias towards accessibility and quality of care in certain areas. Even so, the probabilistic method used is considered to be representative of the results for each city analyzed.

We only included in our sample PHC services that offered TB treatment; health units that did not report TB cases during the study period were excluded. We therefore utilized purposive sampling, which makes it possible to seek depth and situated knowledge about the phenomenon under investigation (Creswell and Creswell, 2022). The fact that some PHC units did not notify TB cases during the study period does not necessarily indicate the absence of the disease, but rather may reflect changes in the surveillance, diagnosis and registration processes [36]. This may indicate underreporting, possibly resulting from overloaded health services, reallocation of teams in response to Covid-19, reductions in active search activities, or the population’s limited access to health services [37].

To be considered as key informants, we only included professionals in the PHC service (indicated either by the management or the team itself) who were directly involved in TB-related activities during the study period.

### Data variables and data collection methods

The quantitative approach utilized a structured form containing 60 questions about the characteristics of the health units and their activities related to TB. This questionnaire was developed by researchers specializing in the area, state and municipal managers of the TB Control Policy, and professionals working in PHC in different scenarios, based on the Manual of Recommendations of the National Tuberculosis Control Program (PNCT) [32]. During the qualitative stage, a semi-structured script (in Portuguese) was used to explore the perspectives of the informants on how the 2020–2022 pandemic period affected provision of TB-related activities as well as the challenges faced during this period. The qualitative sample was defined using the data saturation technique, with an independent evaluation carried out by two researchers who were members of the study team [38].

### Data collection process – quantitative and qualitative

The participants’ contact information was provided by the managers of the health units after their first institutional contact, with prior authorization. They were then contacted by telephone, email or WhatsApp, when the objectives of the study were explained. Data were collected in person, by appointment. No participants refused to participate or withdrew from the data collection process. The study was approved by our institutional review board (case number 5.671.976) and the study participants all signed free and informed consent agreements.

One potential bias in selecting the interview participants is the fact that the contacts were provided by the managers of the health units. This form of access may have affected participant responses, since the managers may have (intentionally or unintentionally) recommended professionals with whom they had a relationship or individuals they considered most representative of the team, which may have excluded critical voices or divergent experiences.

In order to minimize social desirability bias, the researchers adopted strategies to promote spontaneity and authenticity in the responses. During the interviews, they emphasized that all experiences and perceptions were equally important for the purposes of the study. The interviewers were careful to offer a welcoming, empathetic and non-judgmental setting so participants could feel comfortable sharing their experiences without reservations. This approach helped create a safe listening environment that made it possible to collect more consistent and reflective data on the real experiences of the informants. Data collection focused mainly on aspects related to the repercussions of the Covid-19 pandemic on TB-related activities, and took place between October 2022 and January 2023. Quantitative data were collected using the Research Electronic Data Capture (REDCap) tool installed on the researcher’s smartphone. The qualitative data were collected via recorded audio interviews using a semi-structured script. The average time taken to complete the form was 25 minutes, while the interviews lasted an average of 30 minutes. To ensure participant confidentiality, each informant was identified by the letter ‘E’ followed by the numerical order of the interviews.

### Data analysis – quantitative and qualitative

The PHC services were described using descriptive statistical analysis in each city. The TB-related activities that underwent changes were identified based on mean and standard deviation. The study variables relating to these activities were scored using a Likert Scale, with 0 indicating no change and values from 1 to 10 representing different degrees of change in provision of TB-related activities. The quantitative data were analyzed using the Stata statistical software package, version 17 (StataCorp, College Station, TX, USA).

All the interviews were conducted in Portuguese, recorded, and transcribed in full by members of the research team. Excerpts from the interviews were translated into English by a professional translator for inclusion in this article. Once the qualitative data were transcribed, they were organized in Excel spreadsheets (Microsoft, Redmond, WA, USA) to assess saturation [33] and then exported to Atlas.ti software (ATLAS.ti Scientific Software Development GmbH, Berlin, Germany) for analysis. We utilized thematic-categorical content analysis [39], in the analytical approach, which involved pre-analysis, exploration of the material, treatment of the results, inference and interpretation. The research team skimmed the transcripts to determine completeness, representativeness, homogeneity, pertinence and exclusivity.

The data were integrated using the Pillar Integration Process (PIP), a joint display technique that facilitates clear and concise visual presentation of quantitative and qualitative findings. This process involves four stages: listing, matching, verification and construction of the ‘pillars’ or themes. The authors who developed the PIP suggest using a diagram that clearly presents the main inferences in the center, summarizing the integrated findings and showing how they relate to the main themes [40].

## Results

The study involved 207 PHC units: 80 in São Paulo, 47 in João Pessoa, 32 in Florianópolis and 48 in Goiânia. The overall average age of the interviewed informants was 45 years. The sample was predominantly female across all municipalities, with Goiânia standing out with 100% of the participants identified as women. In terms of professional category, 95% of the interviewees were nurses, and in both Goiânia and João Pessoa, all the respondents pertained to this category. The remaining 5% included physicians, nursing technicians, community health agents or other professionals. The average length of experience in PHC among the participants was approximately 2.9 years. The predominant health care model was the family health strategy, used in 90.6% all health units in Florianópolis, 89.4% in João Pessoa, 79.2% in Goiânia and 53.8% in São Paulo. During the study period, changes in work processed during the pandemic were identified in 87% or more of the units in all the cities studied. Absences among medical professionals were reported in all cities, with the highest percentage of units affected in Florianópolis (62.5%) and varying from 29.0% to 34.0% in the remaining three. At the same time, Florianópolis provided the most telemonitoring for TB (in 87.5% of the centers), followed by Goiânia (64.6%), São Paulo (55.0%) and João Pessoa (42.5%). Continuing education offerings varied heterogeneously among the cities and during the study period, with the most offered in São Paulo (in over 52.0% of the centers and peaking at 86.0% in 2022), followed by João Pessoa, with percentages between 17 and 46%. The lowest percentages were seen in Florianópolis (12 to 17%) and Goiânia (6.3 to 17%).

Table 2 presents the rates of change in TB-related activities in the four Brazilian cities during the study period. São Paulo showed the lowest levels of change in all years, with mean values below one for six of the nine activities investigated; still, in 2020 and 2021 it recorded the highest mean rates of change for medication supply and distribution, with values of 3.37 (D: 4.16) and 2.57 (SD: 3.72), respectively, as well as for DOT offered, with averages of 4.46 (SD: 4.11) in 2020 and 3.21 (SD: 3.69) in 2021.

**Table 2.** Rates of change in tuberculosis-related activities offered during the study period (2020–2022) in São Paulo, João Pessoa, Florianópolis and Goiânia, presented as means and standard deviation.

	2020	2021	2022	2020	2021	2022
MEAN (SD)	$\overset{ˉ}{X}$ (SD)	$\overset{ˉ}{X}$ (SD)	$\overset{ˉ}{X}$ (SD)	$\overset{ˉ}{X}$ (SD)	$\overset{ˉ}{X}$ (SD)	$\overset{ˉ}{X}$ (SD)
**CHANGE IN TB-RELATED ACTIVITIES**	**SÃO PAULO**			**JOÃO PESSOA**		
Consultations offered to people undergoing TB treatment	**1.05**2.59	0.72 (2.01)	0.12 (1.01)	1.38 (2.91)	1.36 (2.91)	1.08 (2.61)
Sputum testing offered to assess TB treatment response	1.37 (2.81)	1.11 (2.51)	0.40 (1.39)	2.12 (3.70)	1.59 (3.19)	0.91 (2.70)
HIV testing offered	0.32 (1.49)	0.18 (1.11)	0.11 (1.00)	0.91 (2.70)	0.76 (2.55)	0.38 (1.84)
HIV testing performed	0.28 (1.50)	0.12 (0.80)	0.07 (0.67)	1.08 (2.89)	0.93 (2.76)	0.55 (2.15)
Blood glucose testing offered	0.28 (1.27)	0.18 (1.04)	0.07 (0.56)	0.65 (2.22)	0.65 (2.22)	0.65 (2.17)
Liver function testing offered	0.42 (1.69)	0.18 (1.10)	0.12 (1.01)	1.27 (2.93)	1.27 (2.93)	0.93 (2.54)
Kidney function testing offered	0.42 (1.69)	0.18 (1.10)	0.12 (1.01)	0.93 (2.54)	0.93 (2.54)	0.80 (2.42)
Drug dispensing offered	3.37 (4.16)	2.57 (3.72)	0.81 (2.23)	1.21 (2.89)	1.44 (3.09)	0.53 (2.02)
DOT offered	4.46 (4.11)	3.21 (3.69)	0.63 (1.94)	1.46 (2.99)	1.19 (2.77)	0.76 (2.31)
Chest X-ray offered	0.85 (2.18)	0.63 (1.85)	0.20 (1.16)	1.23 (2.99)	1.23 (2.99)	0.73 (2.15)
	**FLORIANÓPOLIS**			**GOIÂNIA**		
Consultations offered to people undergoing TB treatment	**2.53** 3.58	1.56 (3.16)	0.65 (2.04)	4.39 (4.30)	3.93 (4.07)	1.79 (2.84)
Sputum testing offered to assess TB treatment response	1.34 (2.74)	0.96 (2.46)	0.53 (1.52)	3.89 (4.17)	3.5 (3.90)	1.72 (2.77)
HIV testing offered	0.59 (1.81)	0.34 (1.49)	0.21 (0.87)	3.06 (4.09)	2.95 (3.97)	1.43 (2.68)
HIV testing performed	1.34 (3.09)	0.53 (1.41)	0.21 (0.75)	3.12 (4.14)	2.97 (3.99)	1.41 (2.69)
Blood glucose testing offered	1.06 (2.67)	0.59 (2.07)	0.21 (0.87)	2.54 (3.85)	2.39 (3.72)	0.89 (2.02)
Liver function testing offered	1.28 (2.63)	0.68 (1.71)	0.31 (0.99)	2.79 (3.91)	2.64 (3.79)	1.22 (2.54)
Kidney function testing offered	1.25 (2.56)	0.68 (1.71)	0.31 (0.99)	2.58 (3.79)	2.45 (3.68)	1.02 (2.19)
Drug dispensing offered	0.84 (2.21)	0.71 (2.14)	0.68 (2.08)	2.72 (3.90)	2.27 (3.58)	0.77 (2.08)
DOT offered	2.61 (3.63)	1.67 (3.10)	1.16 (2.69)	3.37 (4.49)	2.60 (3.59)	1.06 (2.20)
Chest X-ray offered	0.65 (1.97)	0.37 (1.26)	0.12 (0.49)	2.50 (3.91)	2.54 (3.80)	1.10 (2.53)

Source: The authors, 2024.

A significant change in treatment activities was observed in Goiânia, especially in 2020 and 2021, when the mean rate of change was 2.5 or more for all the treatment activities analyzed; here, the change in consultations offered to people undergoing TB treatment exceeded the mean rates of change in the other cities, reaching 4.39 (SD: 4.30) in 2020 and 3.93 (SD: 4.07) in 2021. Also notable was the rate of change in offers to request sputum testing for TB treatment response, with a mean of 3.89 (SD: 4.17) in 2020 and 3.5 (SD: 3.9) in 2021.

In João Pessoa and Florianópolis, the mean rates of change were below one for three activities during the entire study period, most notably the offer of HIV testing. In João Pessoa, the activity with the highest mean rate of change was the offer of sputum testing to monitor TB treatment response, at 2.12 (D: 3.70) in 2020, followed by DOT with 1.46 (SD: 2.99). In Florianópolis during the same year, the greatest change was seen in consultations offered to people undergoing TB treatment, with 2.53 (SD: 3.58), and DOT, with 2.61 (SD: 3.63).

Changes in TB-related activities were recorded in all the cities during the study period, to a greater degree in 2020 and to a lesser extent in 2022. Goiânia had the highest mean rate of change, while São Paulo had the lowest for most of the activities we analyzed.

The qualitative stage of the study involved 69 key informants: 24 in São Paulo, 21 in Goiânia, 13 in João Pessoa and 11 in Florianópolis. Table 3 shows the 17 codes generated in the Atlas.ti software, along with the respective frequencies with which they occurred in each city; the codes were grouped into four categories based on thematic similarity.

**Table 3.** Thematic codes tracked in Atlas.Ti software in the four cities studied.

Codes	Frequency codes occurred in each scenario				
	Florianópolis (11 participants)	Goiânia (21 participants)	João Pessoa (13 participants)	São Paulo (24 participants)	Categories
Increased workload due to Covid-19	11	12	9	11	Working conditions
Insufficient staffing	5	8	2	1	
Absences among health professionals	7	19	5	3	
Monitoring of people with TB continued, but with changes to frequency of consultations	3	1	0	5	Care for people with TB
Remote consultations/services	6	0	0	6	
Interruption of home visits	4	0	2	2	
Decrease in user demand for TB care/treatment activities	3	3	1	9	
Damage to contact/relationship with people undergoing TB treatment	2	2	0	4	
Reorganization of workflow at health unit to maintain TB-related in-person care	0	0	0	2	
Changes to medication dispensing	2	0	1	4	Medication dispensing and DOT
TB treatment maintained with no mention of changes or adaptations	4	5	7	3	
Interruption of treatment	1	4	0	2	
Interruption or alteration of directly observed therapy (DOT)	4	1	1	12	
DOT maintained	0	2	1	5	
Supervision of TB treatment by telemonitoring	1	0	2	4	
Difficulties/impairments in monitoring/surveillance of TB progression	2	2	1	0	Monitoring and treatment response testing
Treatment response testing maintained	0	0	0	2	

Source: The authors, 2024.

The following categories were constructed based on our interpretations of the qualitative data.

### Working conditions

During the Covid-19 pandemic, working conditions in PHC units changed significantly in response to measures to ensure continuity of essential services. At the same time, the demand for care soared as a result of the public health emergency, leading to an increased workload and greater pressure on health professionals, as well as illness and sick leave. These factors reduced the availability of human resources for health care.

At first it was very difficult, because there were ten or twelve employees [out] with Covid at the same time. […] It was a huge loss… it put a lot of strain on the team because they weren’t replaced. I myself got Covid twice, so did my [nurse] technician, so did my doctor. I could see that when one of them got it, after about ten days there were six or seven with Covid as well. E7G

### Care for people with TB

The redirection of resources and overloading of health systems led many PHC units and specific TB programs to reduce or halt their regular activities:

[…] It changed our entire work routine, we were basically only involved with Covid and it negatively affected [other] demands, it hindered the service as a whole and created major pent-up demand in all areas, we essentially stopped the programs and only stayed with the routine because all the rest was changed […] E12JP

Treatment […] became a little more complicated because there was a (local) resolution, a directive from the (municipal health) department for these patients to come to the health unit as little as possible. […] So they had their appointments over the phone […] E5SP

Social isolation measures also created barriers to accessing health facilities, which resulted in a considerable reduction in the number of consultations and TB treatment follow-up. Fear of Covid infection also led many users to avoid going to the health units, including for routine consultations and to continue their TB treatment.

[…] patients undergoing treatment were also afraid of catching Covid, of coming to the unit to get their medication and getting Covid, that was the impact… E11SP

Some participants expressed the need for adaptations for people with TB to ensure continuity and effectiveness of care activities:

We did a lot of care via WhatsApp…. we had this means of communication via WhatsApp, but of course for the priority groups (…) we didn’t stop providing service […]. We continued with the tuberculosis patients […]. Maybe not everyone was able to have face-to-face consultations, but the dispensing of medication was not affected, […], we maintained the minimum follow-up, […] even if it was by WhatsApp…. We did a lot of online consultation during the pandemic. E7F

The relationship between the health service and the user being treated for TB is fundamental to ensure adherence to treatment by offering continuous support, enabling recovery and cure and ultimately contributing to control and eradication of this disease. However, the health professionals noted that this relationship changed significantly during the pandemic.

We try to create a good bond with tuberculosis patients so they can truly express what they are feeling and going through. Because sometimes what happens is that they start treatment, they feel well, and they… Some patients think they don’t need to do it anymore, so this bond during the pandemic was a little more difficult for new patients […] those new patients who came in, how were we going to create this bond? Because we build this bond every day, when they come to take their medication. E8SP

Another difficulty identified was the interruption of home visits (to monitor treatment as well as to conduct active searches), which compromised the continuity of care and impeded diagnosis of new cases.

The health workers weren’t making home visits. So we lost a lot in terms of active patient search, even though most [patients] do seek care […] because their symptoms persist, but I think we’ve lost a lot in this sense. E2F

[…] Home visits are very important, and we couldn’t visit in order to not infect [patients][…] so we avoided them. […] we communicated more via cell phone, so that was damaging. E9JP

### Drug dispensing and DOT

The repercussions of the pandemic on treatment activities in terms of medication dispensing and DOT varied in the cities studied as well as among health units in the same city. Different adaptations were also adopted in response to the pandemic emergency. Some participants highlighted the adoption of specific strategies for dispensing drugs (like extending the interval between medication pick-up or providing the medications to family members) to reduce the risk of exposure to Covid-19.

They came to the unit to pick up their medication or had a family member come pick it up, so right from the start of the pandemic we were careful to protect these patients from exposure [that might result from] coming to the unit. E5SP

In several PHC services, the pandemic affected DOT by interrupting direct observation or causing changes in the frequency of supervision (extended to weekly or fortnightly intervals instead of daily). Another adaptation mentioned by the informants was supervision of treatment via telemonitoring.

These patients in observed therapy, the health department asked them to come to the unit only once a week to take their medication, and for more than six days’ supply to be provided so they wouldn’t have to come here every day to take their medicine. E10SP

It became more difficult, but we were still able to maintain control and have them take their medication by means of telemonitoring. E3F

Additionally, in certain contexts the pandemic caused interruptions in the supply chain for drugs to treat TB.

[…] there were times when we had some difficulties with medication… […] there was a delay in our receiving these drugs. E4JP

On the other hand, other participants reported that TB treatment was maintained during the pandemic period in the health units responsible for therapeutic follow-up:

The treatments were carried out normally, right now I can’t recall any significant changes that had any impact on patient care. E6SP

There was no negative impact on people already diagnosed and undergoing treatment. Follow-up for all the appointments at the unit was maintained. E2G

### Monitoring and treatment response testing

Most of the health professionals did not mention this aspect in their statements, with the exception of informants in Florianópolis.

We lost a lot in terms of tuberculosis patient monitoring during that period, we’re trying to get it back now. […] As for treatment, if we couldn’t carry out proper surveillance, proper monitoring of this patient, we must have lost a lot in terms of adherence, even when cases were closed due to cure, or abandonment, this certainly left a lot to be desired. E11F

In São Paulo, some participants said they continued to conduct treatment response testing, despite the difficulties imposed by the pandemic.

[…] they continued to get medical care. Medical monitoring, medication, requesting tests if necessary. So in this type of monitoring, there was no loss. E3SP

We continued to do what we had to do in terms of sputum smears and treatment response X-rays, and we didn’t lose any of the ones we already had. E20SP

However, it is important to note that in Goiânia, one informant reported a lack of specialists to monitor severe TB cases during the pandemic period.

[…] With the pandemic, anywhere you went you couldn’t get a CT scan […] and that part was very complicated. The doctor would refer the patient to a pulmonologist and it would take 6 to 8 months for a slot to open up, because the pulmonologists were all busy with Covid. So all the other lung problems took a back seat, including tuberculosis. E17

After presenting the quantitative and qualitative data separately, Table 4 combines the quantitative findings and the themes that emerged from the statements analyzed in the qualitative approach, highlighting the data integration process. Four main themes emerged from this integration: Consultations within the context of health reorganization; Compromised testing and surveillance; Drug dispensing via collaborative activities; and Changes in directly observed therapy (DOT).

**Table 4.** Integration of qualitative and quantitative data.

TUBERCULOSIS-RELATED ACTIVITIES				
QUANTITATIVE Data	QUANTITATIVE Categories	Main themes	QUALITATIVE Categories	QUALITATIVE Codes
**Changes in consultations offered to people undergoing TB treatment** Goiânia = 3.37 Florianópolis = 1.58 São Paulo = 0.63 João Pessoa = 1.27	Heterogeneity in mean rates of change for consultations offered. Highest rate of change in Goiânia and lowest in São Paulo.	1. **Consultations for people undergoing TB treatment within the context of reorganization of local health systems**	Care for people with TB	’[…] became a little more complicated because there was a (local) resolution, a directive from the (municipal health) department for these patients to come to the health unit as little as possible, […] so they had their appointments over the phone […]’. E5SP
**Changes in sputum testing offered for TB treatment response monitoring** Goiânia = 3.04 Florianópolis = 0.94 São Paulo = 0.96 João Pessoa = 1.54	Heterogeneous rates of change in sputum testing offered. Similar mean rates of change in São Paulo and Florianópolis.	**2. Compromised testing, surveillance and monitoring of people receiving care for TB**	Monitoring and treatment response testing	‘[…] As for treatment, if we couldn’t carry out proper surveillance, proper monitoring of this patient, we must have lost a lot in terms of adherence, even when cases were closed due to cure, or abandonment, this certainly left a lot to be desired.’ E11F ’[…] they continued to get medical care. […] requesting tests if necessary. So in this type of monitoring, there was no loss.’ E3SP ’[We continued] to do what we had to do in terms of sputum smears and treatment response X-rays, and we didn’t lose any of the ones we already had.’ E20SP
**Changes in HIV testing offered** Goiânia = 2.48 Florianópolis = 0.38 São Paulo = 0.20 João Pessoa = 0.68 **Changes in blood glucose testing offered** Goiânia = 1.94 Florianópolis = 0.62 São Paulo = 0.18 João Pessoa = 0.65 **Changes in liver function testing offered** Goiânia = 2.22 Florianópolis = 0.76 São Paulo = 0.24 João Pessoa = 1.16 **Changes in kidney function testing offered** Goiânia = 2.02 Florianópolis = 0.75 São Paulo = 0.24 João Pessoa = 0.89	Goiânia had the highest mean rates of change in testing offered (blood glucose, kidney function, liver function). Florianópolis and São Paulo had averages below 0.75.			
**Changes in chest X-rays offered** Goiânia = 2.04 Florianópolis = 0.38 São Paulo = 0.56 João Pessoa = 1.06	Goiânia had the highest mean rate of change for chest X-rays offered, followed by João Pessoa. São Paulo and Florianópolis had the lowest rates.			
**Changes in medication dispensing offered** Goiânia = 1.92 Florianópolis = 0.74 São Paulo = 2.25 João Pessoa = 1.06	São Paulo showed the greatest change in drug dispensing offered, followed closely by Goiânia. Change in João Pessoa was intermediate, followed by the lowest rate of change in Florianópolis.	**3. Maintaining drug dispensing via collaborative activities to reduce exposure of people undergoing treatment for TB**	Medication dispensing and DOT	‘They came to the unit to pick up their medication or sent a family member to pick up the medication, so right from the start of the pandemic we were careful to protect these patients from exposure [that might result from] coming to the unit.’ E5SP
**Changes in DOT offered** Goiânia = 2.34 Florianópolis = 1.81 São Paulo = 2.76 João Pessoa = 1.13	The mean rates of change were highest in São Paulo, followed by Goiânia. Florianópolis and João Pessoa recorded intermediate rates of change.	**4. Change in frequency of DOT within the context of reducing infection risk**		“These patients in observed therapy, the health department asked them to come to the unit only once a week to take their medication, and for more than 6 days’ supply to be provided so they wouldn’t have to come here every day to take their medicine.” E10SP

**Source**: The authors, 2024.

## Discussion

This study is a pioneering investigation into the repercussions of the Covid-19 pandemic on TB care and management activities in four Brazilian cities. Brazil’s PNCT is internationally recognized as one of the best structured plans for addressing tuberculosis, from a technical, organizational and operational point of view [32,41]. But even despite this structure, TB-related services were significantly impacted throughout the country during the pandemic, mainly in terms of operational processes [21].

The integration of the quantitative and qualitative results in this mixed-method study revealed changes in how TB treatment was provided in PHC services in the four cities studied during the pandemic. This finding corroborates work by Coutinho et al. [23] and Ribeiro et al. [42], which reported interruptions and other complications in TB care services around the world during this period.

In our study, the greatest repercussions on TB-related activities were observed in Goiânia and João Pessoa, which may be related to PHC’s capacity to cope with the pandemic, in addition to economic and social aspects such as inequality in access to health services, population concentration in vulnerable urban areas, unemployment and low levels of education [43].

Although Brazil has the world’s largest public health system, with universal access and an extensive PHC network that plays an important role in TB care and treatment, significant bottlenecks persist in various areas such as financing, management, human resources and structuring of services [44,45]. In 2017, Brazil’s National Primary Care Policy was revised to implement changes in team configuration, financing and accreditation [42]. The new resulting work dynamic, combined with structural limitations and challenges related to work processes, may have compromised TB management activities [46–48]. Furthermore, the lack of government policy at the federal level and neglect resulting from pandemic-related efforts during the study period exacerbated the deleterious effects of the health crisis in certain contexts [49].

Studies prior to the pandemic showed limitations and weaknesses in the PHC work process that compromised TB management activities [47,48]. Even so, it is undeniable that the care dynamics in PHC for people with TB were transformed in various aspects, mainly due to the major changes that occurred overnight in response to the pandemic; these changes demanded restructuring of services and ultimately reduced the health system’s capacity to maintain the supply of services [19,47,50]. Additionally, quarantine and restrictions on movement [48] further affected this scenario, as our findings show (namely in the first theme in the data integration, as shown in Table 4).

Brazil’s response to the pandemic occurred within a context marked by divergent institutional guidelines resulting from a lack of national coordination [51,52]. This led to heterogeneous definition of priorities and the implementation of policies by state and municipal managers [53].

In the city of Goiânia, TB-related activities changed the most after a municipal decree in March 2020 declaring a state of emergency due to the Covid-19 pandemic; this ordinance reassigned medical professionals to emergency services [54]. The schedules of physicians and nursing professionals in PHC were also suspended to ensure that 70% of appointments would be available for priority care of people with respiratory symptoms suggestive of Covid-19 [54]; only 30% was reserved for all other PHC programs and activities, including those related to TB [55]. The scenario may have been similar in the other cities we studied, if perhaps in different proportions and with significant variations in the provision of TB-related activities.

The first data integration theme, ‘Consultations for people undergoing TB treatment within the context of reorganization of the local health system,’ highlights the central nature of this activity in the treatment process [31,52], especially considering that the mortality rate for untreated TB can reach approximately 50% [1,5]. Treatment has a cascading effect on care, since treatment activities are linked and take place during the patient’s consultations [26].

During the consultation, barriers and difficulties that might affect adherence to treatment, therapeutic effectiveness and cure should be identified. This is also an opportunity to alter medication dosage, assess clinical progress based on treatment control testing (bacteriology as well as X-ray), screen for TB/HIV co-infection and order complementary testing of blood glucose and liver or kidney function, when necessary [30]. Consults also provide a chance to promote guidelines that boost adherence to the treatment regimen [26]. Lack of adequate follow-up can result in unfavorable outcomes, such as treatment interruption or failure and, in more serious cases, death [56].

The second theme, ‘Compromised testing, surveillance and monitoring of people receiving care for tuberculosis,’ may be related to the shortage of human resources. Some studies have shown a reduction in staffing during the pandemic, aggravated by sick leave, risk group status, and reallocation of professionals [19,20,57,58]. Data from the Brazilian Ministry of Health indicate that 26,555 nurses (14.7%) and 19,858 doctors (11.02%) were affected by Covid-19 [59]. Contact between health professionals and people undergoing TB treatment was complicated not only by changes in the organization and work process, but also by rules related to isolation and social distancing [30]. These rules limited user access to health services for fear of exposure to the SARS-CoV-2 virus [60] and limited home visits.

One study conducted in southern Brazil found that despite an increase in laboratory demand, there was a reduction in the supply of TB services, especially in PHC [61]. Although testing resumed at different rates throughout the pandemic in the cities we investigated, it is important to consider that activities are reinstated gradually amid a health emergency, and are subject to the local dynamics as health services are reorganized. Sputum microscopy, which is essential to verify the effectiveness of TB drug treatment, exemplifies the importance of regular exams, but this and other testing may have been impeded by logistics-related obstacles related to ordering and collection of samples.

Testing plays an important role in health services, especially during TB treatment, given that a person living with HIV has a risk of developing active TB 28 times higher than the general population [58], and also considering that rates for ordering and performing HIV tests were low even before the pandemic [62,63]. It is also important to remember that when TB is associated with diabetes, high blood glucose levels can compromise the effectiveness of anti-tuberculosis drugs, and higher doses of TB drugs can reduce the effectiveness of medications used to treat diabetes [64]. Furthermore, certain anti-tuberculosis drugs can cause liver toxicity, which may be exacerbated by drug interactions or doses that exceed recommendations [30]. These aspects reinforce the importance of regular laboratory and imaging tests throughout TB treatment, and highlight the need to resume and intensify these practices after the pandemic, with the support of strategies to overcome barriers that still exist in health services. The changes in chest X-rays offered that we observed are likely the result of structural problems that existed prior to the pandemic. Some cities only have diagnostic support (radiology services) in urgent and emergency care units, and outsource radiology services for PHC. Such a configuration likely contributed to the continued and even increased presence of barriers during the pandemic. One study in northern Brazil found that fewer chest X-rays were performed on people suspected of having TB in public health services during the pandemic [14]. Another study in southern Brazil found that during the pandemic, the least frequently offered TB-related activities were three or more sputum microscopy tests for treatment response, sputum microscopy at the end of treatment, chest X-rays in the sixth month and sputum culture; the number of medical and nursing consultations did not reach the minimum of six recommended visits [65].

In general, TB surveillance activities were also impacted during the pandemic. A scoping review identified a significant reduction in TB case notifications, closure of cases due to cure, the number of people starting treatment, and case follow-up. There was also an increase in the number of deaths, patients undergoing retreatment, loss to follow-up of individuals with resistant TB, and interruption of DOT during the pandemic [42].

With this in mind, financial investments in the public health system with an emphasis on PHC are needed to ensure adequate funding for diagnostic support in health units, reducing dependence on outsourced services. We should note that maintaining contracts with outsourced services during the pandemic may have been a challenge, given the high costs municipal governments faced in response to the pandemic emergency [66].

São Paulo stood out among the cities we analyzed for its high mean rate of changes related to the third theme, ‘Maintenance of drug dispensing with collaborative activities to reduce exposure of the people undergoing treatment,’ showing that the supply of TB drugs in health services was sustained during the pandemic. Local management authorized delivery of medications prescribed for continuous use in São Paulo [67] and Goiânia [51], which maintained the monthly flow of TB drugs. Within this context, self-administered treatment was recommended for adults and people with TB/HIV co-infection in a basic regimen as a strategy to reduce exposure to SARS-CoV-2 [67]. Similar findings were identified in the literature around the pandemic’s impact of TB drug dispensing in terms of reduced supply and drug pick-up [9,11] as well as longer intervals between drug dispensing [10]. These changes may be associated with various factors such as the reorganization of health services, changes to the flow of care for people undergoing treatment, absences among health professionals and changes to drug dispensing routines during the pandemic.

The aspects addressed in the second theme also underscore the findings for the fourth theme, ‘Change in frequency of DOT within the context of reducing infection risk.’ While DOT contributes to adherence to TB treatment, this strategy was not viable during the health emergency [11]. One international study documented this change in other countries like Portugal, where even though health services continued during the pandemic there were delays in diagnosing TB and conducting DOT [68].

The city of São Paulo had the highest mean rate of change in DOT provided among our sample; this change is related to the guidelines of the Municipal TB Control Program, which at the beginning of the pandemic limited DOT to certain priority groups like homeless people, IV drug users or patients on a regimen for resistant TB [64]. The offer of supervised treatment five times a week for the entire population only resumed in September 2021 [69]. During this period, São Paulo’s city government reinforced alternative strategies such as the use of technology to monitor therapy, as well as assigning professionals who lived close to people undergoing treatment to be responsible for supervising patients as they took their medication [64]. However, the change in this activity may have led to losses, as pointed out in the literature: loss of follow-up, an increase in users undergoing retreatment and an expanded period of TB treatment [8,14].

It is important to note that vaccination against Covid-19 was prioritized in Brazil as of January 2021, which kept the other programs carried out in the PHC setting in limbo until the vaccines were applied [70]. Although the average changes in TB treatment activities diminished over the course of the study period, health professionals were overwhelmed by health services overburdened with confirmed and suspected Covid-19 cases as well as the logistical and operational demands related to the vaccination campaign [11,71].

Another notable factor is the heterogeneous nature of FHS coverage in the cities we studied, which along with the limited continuing education offerings on TB during the pandemic period may have compromised PHC’s ability to respond to Covid-19, consequently impacting TB treatment activities. One national cohort study found that higher FHS coverage was associated with lower TB incidence and mortality rates, as well as higher cure rates [72]. On the other hand, a literature review found a significant allocation of investment in hospital care and the private sector in Brazil, to the detriment of PHC’s potential as a strategic component of the SUS [73].

### Strengths

This study was conducted during the pandemic, and its findings reflect the realities observed within the cities and health services we analyzed during this period. The use of mixed methods (still unprecedented in this area) permitted comprehensive assessment of the repercussions of the Covid-19 pandemic on TB treatment and monitoring activities. The team responsible for the qualitative stage of the study had expertise in applying mixed methods, which helped ensure methodological quality, data integration and objectivity in analyzing and interpreting the findings. The cities we investigated are state capitals with large populations, making the results relevant and representative. We adopted the Strengthening the Reporting of Observational Studies in Epidemiology (STROBE), Consolidated Criteria for Reporting Qualitative Research (COREQ) and Mixed Methods Appraisal Tool (MMAT) guidelines to report quantitative and qualitative components, respectively [74,75].

### Limitations

The study was carried out in four Brazilian state capitals, which limits its ability to be generalized to other contexts. The reality in cities with lower HDI scores, reduced FHS coverage, fewer primary health units and higher TB incidence rates may differ from what we observed in this study. Moreover, specific local policies on care for people with TB may have influenced the results, since each city had its own approaches to address the challenges resulting from the pandemic.

### Future study

Some relevant aspects were not considered in this research, and could be explored in future efforts. This study did not include managers, representatives of local health councils or local TB committees, or people undergoing TB treatment. Including these actors in future studies could provide a more comprehensive understanding of institutional dynamics, local governance strategies and the experiences of health service users during the pandemic period.

### Implications for the program

Based on the literature and the findings of this research, some relevant implications for improving the National Tuberculosis Control Program can be identified. These recommendations include: (1) Developing permanent education strategies related to work during health emergencies, as well as effectively incorporating telemonitoring as a complementary tool in TB care; (2) Implementing an information system linked to the National TB Control Program that can process diagnostic and treatment data in real time, especially during emergencies, based on consolidated experiences [76]; (3) Creating easily accessible dashboards that permit real-time monitoring of TB care activities; (4) Documenting the challenges faced during the process of resuming activities after the pandemic, and evaluating the implementation of strategies and their qualification within the work process.

## Conclusion

This study examined the impact of the Covid-19 pandemic on TB-related activities in four Brazilian state capitals between 2020 and 2022, as described by informants within health units offering this care. We identified barriers related to the reorganization of health services, reduced supply of consultations and tests, limitations in surveillance and case monitoring, as well as discontinuation of DOT; we also presented mechanisms that contributed to these impacts, such as local ordinances and guidelines and a lack of national coordination. While some alternative strategies for consultations and follow-up were cited (such as teleconsulting), these strategies require systematic evaluation to determine their effectiveness and impact on quality of care. Testing was particularly impacted, highlighting the need to evaluate laboratory networks, availability of supplies and professionals, quality of care provided (especially with regard to ordering and carrying out tests) and difficulties that impede treatment such as transportation costs. And while reorganization of health services is expected during health emergencies, it is essential to develop specific protocols to ensure that TB surveillance activities continue in future scenarios. Important and recurring themes were communication with the public, continuous training of health professionals, and follow-up and systematic evaluation of activities, along with tuberculosis literacy. Addressing these barriers can help improve the response of health services during future crises in order to mitigate impacts, increase quality of care, and make progress in fighting and eliminating TB.

## Data Availability

The data used in this study are available upon request from the corresponding authors, in accordance with ethical criteria for sharing data.
